# *In vitro* anti-TB properties, *in silico* target validation, molecular docking and dynamics studies of substituted 1,2,4-oxadiazole analogues against *Mycobacterium tuberculosis*

**DOI:** 10.1080/14756366.2021.1900162

**Published:** 2021-06-01

**Authors:** Pran Kishore Deb, Nizar A. Al-Shar’i, Katharigatta N. Venugopala, Melendhran Pillay, Pobitra Borah

**Affiliations:** a Department of Pharmaceutical Sciences, Faculty of Pharmacy, Philadelphia University, Amman, Jordan; b Department of Medicinal Chemistry and Pharmacognosy, Faculty of Pharmacy, Jordan University of Science and Technology, Irbid, Jordan; c Department of Pharmaceutical Sciences, College of Clinical Pharmacy, King Faisal University, Al-Ahsa, Kingdom of Saudi Arabia; d Department of Biotechnology and Food Technology, Durban University of Technology, Durban, South Africa; e Department of Microbiology, National Health Laboratory Services, KZN Academic Complex, Inkosi Albert Luthuli Central Hospital, Durban, South Africa; f Pratiksha Institute of Pharmaceutical Sciences, Guwahati, India

**Keywords:** 1,2,4-oxadiazoles, *Mycobacterium tuberculosis*, multidrug-resistant *Mycobacterium tuberculosis*, molecular docking, dynamic studies, MTB target validation

## Abstract

The alarming increase in multi- and extensively drug-resistant (MDR and XDR) strains of *Mycobacterium tuberculosis* (MTB) has triggered the scientific community to search for novel, effective, and safer therapeutics. To this end, a series of 3,5-disubstituted-1,2,4-oxadiazole derivatives (**3a–3i**) were tested against H37Rv, MDR and XDR strains of MTB. Of which, compound **3a** with para-trifluorophenyl substituted oxadiazole showed excellent activity against the susceptible H37Rv and MDR-MTB strain with a MIC values of 8 and 16 µg/ml, respectively.

To understand the mechanism of action of these compounds (**3a–3i**) and identify their putative drug target, molecular docking and dynamics studies were employed against a panel of 20 mycobacterial enzymes reported to be essential for mycobacterial growth and survival. These computational studies revealed polyketide synthase (Pks13) enzyme as the putative target. Moreover, *in silico* ADMET predictions showed satisfactory properties for these compounds, collectively, making them, particularly compound **3a**, promising leads worthy of further optimisation.

## 1. Introduction

Tuberculosis (TB) is known to infect human beings for thousands of years with cause remained unknown until the year 1882, when Dr. Robert Koch discovered the bacillus- *Mycobacterium tuberculosis* (MTB)^1^^,^^2^. TB is an airborne communicable disease that predominantly affects the lungs (pulmonary TB) but can also disseminate to other sites (extrapulmonary TB). Globally, about 1.2 billion people (a quarter of world population) are infected with MTB that may remain dormant throughout their lifespan, of which 5–10% are at high risk of developing active TB disease, especially the immuno-compromised patients. Notably in 2019, MTB claimed the lives of approximately 1.2 million among the human immunodeficiency virus (HIV)-negative, and 208,000 among HIV-positive people^3^. Since the first availability of effective drug treatments in the 1940s, remarkable global efforts have been made to control the TB related morbidity and mortality. The current recommended treatment for drug-susceptible TB (DS-TB) comprises of 6-month regimen of 4 first-line drugs viz. isoniazid, rifampicin, pyrazinamide and ethambutol^4^. However, drug-resistant TB (DR-TB) continues to be a potential public health threat due to the critical hindrance with the efficacy of existing drug regimens^5^. In 2019, about 465,000 new incidents of rifampicin-resistant TB (RR-TB), including 78% multidrug-resistant TB (MDR-TB) cases – MTB strain conferring resistance at least to rifampicin and isoniazid were reported, with an estimated 182,000 deaths^3^. Moreover, RR-TB/MDR-TB regimen usually comprises of longer, expensive (estimated ≥1000 USD/person) and more toxic drugs with low cure rate (56%), thereby increasing the economic burden as well^6^. These intractable MDR-TB strains also comprises of extensively drug-resistance TB (XDR-TB) – MDR strain with additional resistance towards at least a fluoroquinolone and a second-line injectable drug. Furthermore, daunting scenario of totally drug-resistant TB (TDR-TB) strains in clinical practice is miserable^7^^,^^8^. Presently, 23 drug candidates are under clinical development for the treatment of various forms of TB including latent TB, DS-TB and MDR-TB. Interestingly, 13 of these compounds are novel drugs, of which bedaquiline, delamanid and pretomanid have already been approved by the regulatory authorities^9–11^. Unfortunately, the first resistant MTB clinical isolates against bedaquiline and delamanid was reported within 2 years of their approval^12^. Soon, bedaquiline resistance turns out to be routine in clinical practice, suggesting the limited time window for a new drug^13^. Additionally, bedaquiline and delamanid are also ineffective against TDR-TB^14^. Overall, these issues point towards the compelling need for rapid drug development with a continuous reinforcement of the research pipeline. Therefore, novel, effective, and safer anti-TB drugs are the unmet need not only to overcome the acquired resistance but also to shorten the longer drug regimens that contribute to the nonadherence and subsequent increase in the antibiotic-resistance. To pursue this goal, our experimental efforts are focussed to develop novel compounds from new chemical classes with potent anti-TB activity.

In continuation to our anti-TB drug discovery efforts and in search of novel scaffolds as anti-TB agents, we have designed, synthesised and reported several natural products, cyclic depsipeptides and compounds belonging to various heterocyclic scaffolds such as aminoquinazolines, benzothiazoles, pyrrolo[1,2-*a*]quinolines, dihydropyrimidines, 1–(5-isoquinolinesulfonyl)piperazines, triazoles, triazolyl 1,2,3,4-tetrahydropyrimidines, and various substituted indolizines as potential anti-TB agents^15–25^.

The synthesis of 1,2,4-oxadiazoles (originally azoxime or furo[ab1]diazoles) was first done by Tiemann and Krüger in 1884 – just 2 years after the discovery of MTB^26^. Although this scaffold remained poorly characterised until the early 1960s, their strong tendency of photochemical reactivity and rearrangements have later intensified the interest towards this heterocyclic compound^27^^,^^28^. However, 1,2,4-oxadiazole have also impacted the medicinal chemistry research after the launch of first-in-class marketed drug, oxolamine as a cough suppressant, and the subsequent discovery of commercial products like prenoxdiazine, fasiplon, pleconaril, proxazole, atalureri and butalamine^29–31^. These clinical success in parallel gave an impetus to the synthetic chemists to design innovative strategies for the economic, ecofriendly and rapid synthesis of 1,2,4-oxadiazole chemical libraries to accelerate the drug discovery process^32^^,^^33^. In the past 40 years, 1,2,4-oxadiazole scaffold has been extensively investigated for diverse biological activities^34^ including anti-inflammatory^35^^,^^36^, analgesic^37^, anaesthetic^38^, anthelmintic^39^, antiallergic^40^, anti-Alzheimer^41^, antibacterial^42^^,^^43^, anticancer^44–46^, anticonvulsant^47^^,^^48^, antidepressant^49^, antifungal^50^, anti-HIV^51^, antiparasitic^52^, antiplatelet and antithrombotic^53^, anti-tubercular^54^, antitussive^55^, antiviral^56^^,^^57^, insecticidal^58^, monoamine oxidase inhibition^59^, muscarinic receptor agonists^60^, selective G-protein bile acid receptor 1 (GPBAR1) agonists^61^ and selective H_3_ receptor antagonists^62^ activities.

It has been revealed that some 1,2,4-oxadiazole derivatives may have significant importance in the development of novel anti-TB agents due to the ease of structural modifications. A series of novel styryl-1,2,4-oxadiazoles inspired by the chemical structure of cinnamic acid were evaluated for anti-TB activity against MTB H37Ra strain^63^. Very recently, anti-tubercular activity of a series of 1,2,4-oxadiazoles^64^ and substituted 1,2,4-oxadiazol-3-ylmethyl-piperazin-1-ylquinolones^65^ have also been reported. After extensive literature survey, it has been observed that, till date no studies were conducted to determine the anti-TB activity of 3,5-disubstituted-1,2,4-oxadiazoles. Recently, we have reported the synthesis of a novel series of 3,5-disubstituted-1,2,4-oxadiazoles^43^^,^^66^. In the present study, we report the anti-tubercular activity of these 3,5-disubstituted-1,2,4-oxadiazoles against H37Rv, MDR and XDR strains of MTB. Further, intensive in silico studies were implemented aiming to reveal the putative target of these compounds, hence, explaining their probable mechanism of action.

## 2. Materials and methods

### 2.1. Chemistry

**Scheme 1.**
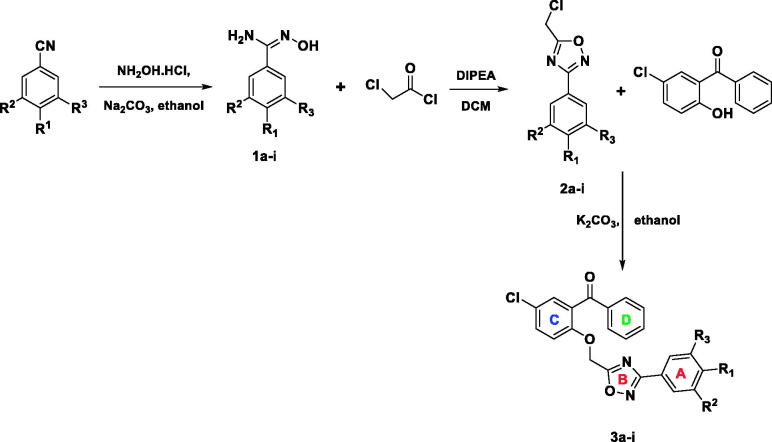
Synthetic scheme of 3,5-disubstituted-1,2,4-oxadiazoles (**3a–3i)**.

Synthesis of title compounds (**3a–3i**) is depicted in Scheme 1 and was achieved according to our previous reports^43^^,^^66^.

### 2.2. Anti-tubercular screening: resazurin microplate assay (REMA)

Anti-TB screening of test compounds **3a–3i** (Table 1) was performed using the colorimetric REMA plate approach^19^^,^^67^. In order to determine the minimum inhibitory concentration (MIC), test compounds (**3a–3i**) were assessed using the agar incorporation approach, which was performed three times, and targeted in H37Rv and MDR-TB strains (isoniazid, 0.2 µg/mL; rifampicin, >1.0 µg/mL). MIC determination was then carried out with some modifications^68^. A Level II Biosafety Laboratory was used to carry out this experiment. MTB reference strain H37Rv (American Type Culture Collection [ATCC], Manassas, VA, USA: 25177) and MDR-TB were cultured in Middlebrook 7H11 medium for a total of 3 weeks^69^. The strain was supplemented with OADC (0.005%, *v/v*, oleic acid; 0.2%, *w/v*, glucose; 0.085%, *w/v*, NaCl; 0.02%, *v/v*, catalase; and 0.5%, 171 *w/v*, bovine serum albumin [BSA]), and incubated at a temperature of 37 °C. Fresh cultures were used to prepare a standardised inoculum in a sterile tube (5 mm in diameter) containing 0.05% Tween 80 and 4.5 ml of phosphate buffer for vortexing. The bacterial supernatant was then standardised to McFarland Number 1 with water, resulting in a bacterial concentration ∼1 × 10^7^cfu/mL. The bacterial suspension was then diluted with water, after which a total of 100 µL of the dilution was placed onto Middlebrook 7H10 agar plates with drug doses ranging from 128–0.125 µg/mL (to begin, 8 µg/mL of the drug was dissolved in distilled water and diluted two fold to achieve the desired concentration prior to being added to the agar medium). The MICs of the drugs (i.e. the concentration that inhibited >1% of the organism’s growth when compared with controls) were obtained 3 weeks following the incubation. These compounds were also tested against previously well-characterised M(XDR) – TB clinical isolates. These isolates were characterised using both a gold standard agar proportion susceptible testing method and a genotyping PCR (MTBDRplus & MTBDRsl, Hain-Lifesciences, Germany). MDR TB clinical isolates were resistant to both isoniazid and rifampicin only whereas XDR TB clinical isolates were resistant to isoniazid, rifampicin, a fluoroquinolone, and an aminoglycoside/cyclic peptide.

**Table 1.** In vitro anti-TB activity of ((5-chloro-2-((3-substituedphenyl-1,2,4-oxadiazol-5-yl)methoxy)phenyl) (phenyl)methanone molecules (**3a–i**) against H37Rv, MDR and XDR strains of *M. tuberculosis.*

Compound ID	Chemical Structure	MIC (µg/ml)		
		Susceptible	MDR	XDR
3a		8	16	No activity
3b		32	32	No activity
3c		11	No activity	No activity
3d		8	32	No activity
3e		32	128	No activity
3f		128	No activity	No activity
3g		32	No activity	No activity
3h		64	No activity	No activity
3i		11.3	No activity	No activity

### 2.3. Computational methods

#### 2.3.1. Computational Materials

The compounds (**3a–3i**) were initially sketched using ChemBioDraw Ultra 12 and were then imported into Discovery Studio (DS) 2017 (BIOVIA, Dassault Systèmes, Discovery Studio, 2017, San Diego: Dassault Systèmes, 2017) for further preparation. All computational procedures including ligand and protein preparation, molecular docking, rescoring, and ADMET predictions were conducted using DS. Molecular dynamics (MD) simulations was performed using Amber12^70^. The virtual molecular dynamics software (VMD) 1.9.3^71^ was utilised to manipulate the generated trajectories. High quality images were generated using PyMol molecular graphics system^72^ and DS.

#### 2.3.2. Workflow

**Figure 1.**
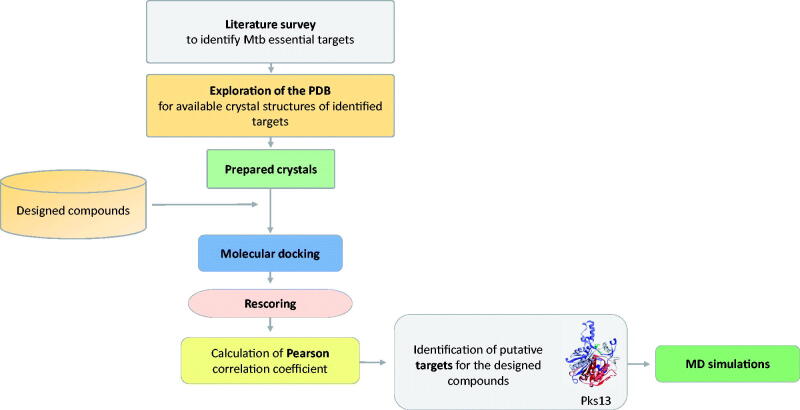
Summary of the workflow to identify putative target for the compounds **3a–3i**.

In order to identify a putative target for the compounds **3a–3i**, extensive literature survey was carried out to identify mycobacterial drug targets deemed essential for bacterial growth and survival. Then, the Protein Data Bank (PDB) repository was explored to obtain 3 D crystal structures for the identified essential targets that will serve as structural models in molecular docking steps. Once identified, all of the compounds (**3a–3i**) were docked into the binding site of each of those targets and were then rescored using different scoring functions. Afterwards, the Pearson correlation coefficient between the experimental MIC values and computational scores of these compounds (**3a–3i**) was calculated. Based on the correlation values, macromolecular targets that showed the highest correlation values were identified as putative targets for the designed compounds. Finally, MD simulations of a virtual complex of the most active compound (**3a**) with its putative target were performed in order to establish a deeper insight of its binding mode and stability with the target enzyme. Figure 1 summarises the workflow applied in this study.

#### 2.3.3. Compounds preparation

All compounds (**3a–3i**) were sketched using ChemBioDraw Ultra 12, imported into discovery studio (DS) and converted into their corresponding 3 D structures using the Prepare Ligand protocol. This protocol assigns proper bond orders, generate different chemical isomers, tautomers and ionisation states. Default parameters were utilised except for the “generate tautomers” which was set to canonical tautomer.

#### 2.3.4. Preparation of crystal structures

The crystal structures of the essential mycobacterial drug targets retrieved from the PDB were checked using Protein Report tool to identify any missing loops, alternate conformations, or incomplete residues. Then they were cleaned and prepared using the Clean Protein tool and Prepare Protein protocol to clean the crystal structures, correct connectivity and bond order, standardise atom names, and protonate proteins at pH of 7.4^73^. All default parameters were used except for keeping ligands and all water molecules which were set to true. Then, the prepared crystal structures were solvated by immersing them in a box of pre-equilibrated TIP3 water molecules using the Solvation protocol. After solvation, they were sequentially minimised in three stages using the Minimisation protocol with default parameters as detailed previously^74–76^. Finally, all water molecules and counterions were deleted, then the binding sites were defined based on the co-crystallized ligands using the Define and Edit Binding Site tool, and then the ligands were deleted. Once the binding sites were defined, the crystal structures were ready to start the docking phase which was commenced using the CDOCKER docking protocol^77^.

#### 2.3.5. Molecular docking and scoring

Prior to docking the designed compounds into the prepared crystal structures, the docking protocol was validated by redocking each of the co-crystallized ligands into their corresponding protein. The accuracy of the docking protocol and the definition of the binding sites were assessed by measuring the ability of the docking algorithm to reproduce the binding orientation of the native co-crystallized ligand via calculating the root mean square deviation (RMSD) between the docked and native pose for each protein complex. Once the docking protocol (CDOCKER) was validated, the designed compounds were docked into the binding site of each of the prepared protein crystal structures using default parameters. Then, the resulting docked poses were rescored using another seven scoring functions, namely LigScore1 and LigScore2^78^, PLP1^79^ and PLP2^80^, Jain^81^, PMF^82^ and PMF04^83^. Finally, putative targets were identified by calculating the Pearson correlation coefficient^84^ between the experimental MIC values and computational docking scores.

#### 2.3.6. Calculation of free binding energy (MM-PBSA)

In order to further examine the binding orientation and binding interactions of the compounds (**3a–3i**) with their putative target, their free binding energies were calculated. The compounds were *in situ* minimised using the In-Situ Ligand Minimisation protocol, using default parameters. Then, their binding free energies were calculated using the Calculate Binding Energies protocol, where the Poisson Boltzmann with non-polar surface area (PBSA) solvent model^85^ was used to account for solvent effects, the ligand conformational entropy value was set to true, and the BEST conformation generation method was applied.

#### 2.3.7. Molecular Dynamics simulations

The top ranked docked pose based on free binding energy scores of the most active compound (**3a**) with its putative target was used as a structural model for running the simulations. Moreover, the apo form of the identified putative target enzyme along with its complexed crystal structure with an inhibitor was also simulated for comparison purposes.

The simulations were run using AMBER 12 as detailed in previous studies^76^^,^^86^. Prior to running the simulations, parameters for the non-standard residue (the most active compound in the designed set, and the co-crystallized ligand) were generated using the Antechamber program of Amber12 utilising the General AMBER Force Field (GAFF)^87^ and AM1-BCC charge method. Briefly, the simulation protocol involved solvation of the simulated systems using TIP3P water, sequential minimisation, heating from 0 to 310 K under constant volume and temperature conditions (NVT ensemble); equilibration for 1 ns under constant temperature and pressure conditions (NPT ensemble); and then the production phase commenced for 100 ns under NPT conditions. Langevin dynamics scheme^88^ under periodic boundary conditions was used with a collision frequency of 2 ps^−1^; the SHAKE algorithm was used to restrain hydrogens^89^ allowing a 2 fs time step; and long-range electrostatic interactions were simulated using the Particle Mesh Ewald (PME) method^90^ with a non-bonded cut-off value of 12 Å. The generated trajectories were then analysed by calculating their thermodynamic properties, RMSD, and root mean square fluctuations (RMSF).

#### 2.3.8. Absorption, distribution, metabolism, excretion and toxicity (ADMET) predictions

Different pharmacokinetic and toxicity parameters for the designed compounds were calculated using the ADMET Descriptors protocol and the Toxicity Prediction (TOPKAT) protocol in DS.

## 3 Results and discussion

### 3.1. Chemistry

Synthesis of 3,5-disubstituted 1,2,4-oxadiazole derivatives (**3a–3i)** was achieved by reacting 5-(chloromethyl)-3-substituted phenyl-1,2,4-oxadiazoles (**2a–2i)** and substituted benzophenone reactants in absolute ethanol medium. Purification of the title compounds was achieved by recrystallization method, and yield was in the range of 83–93% by microwave method of synthesis^66^. Intermediates (**2a–2i)** were synthesised by reacting chloroacetyl chloride and substituted *N*'-hydroxybenzimidamides (**1a–1i)** at equimolar proportion with the help of *N, N*-diisopropylethylamine (DIPEA) in dichloromethane (DCM) solvent. The yield of the intermediates was found to be in the range of 56–75%. The synthesis of *N*'-hydroxybenzimidamides (**1a–1i)** was accomplished from the corresponding substituted benzonitriles and hydroxylamine hydrochloride in methanol medium in the presence of sodium carbonate. The yields of the compounds **1a–1i** were in the range of 58–79%.

### 3.2. Pharmacology

The *in vitro* inhibitory activity of the designed compounds against H37Rv, MDR and XDR strains of MTB was performed as described in the methodology section. Table 1 presents the anti-TB results of the compounds **3a–3i** tested against H37Rv (ATCC: 25177), MDR and XDR strains of *M. tuberculosis*. The *in vitro* results showed excellent inhibitory potency where the MIC values against H37Rv strain were found to be in the range of 8–128 µg/ml, and three compounds **3a, 3 b,** and **3d** exhibited good potency against MDR-MTB strain with MIC values 16, 32, and 32 µg/ml, respectively. It is interesting to note that compounds **3a** and **3d** were the most active against H37Rv strain having the same potency (MIC = 8 µg/ml). However, compound **3a** with para-trifluorophenyl group attached to the 3-position of the oxadiazole ring was found to be the most active against the MDR-MTB strain with a MIC value of 16 µg/ml. None of the compounds (**3a–3i**) showed any activity against the XDR strain.

### 3.3. Computational modeling studies

#### 3.3.1. Identification of putative drug targets for the compounds 3a–3i

During the last two decades, most of the anti-mycobacterial drug discovery efforts were focussed on biochemical, target-based inhibitor screening, which unfortunately did not result in any new TB drugs. Therefore, recent anti-TB efforts have been shifted to the development of whole-cell screening assays, yet, this approach is still quite challenging because of the complex nature of the microenvironments being encountered by the causative agent of TB, *M. tuberculosis,* within the human host. Hence, different screening methods have been developed to better mimic the *in vivo* conditions of MTB inside the bodies of TB patients^91^.

Even with the use of whole-cell screening approaches, once a promising hit or a lead compound is identified, its specific mycobacterial drug target need to be identified in order to guide the prospective optimisation processes towards designing drug-like compounds. In general, an ideal drug target that can be utilised to develop clinically useful antibiotic need to be essential *in vivo*, drug vulnerable, and druggable.

In our current study, the anti-tubercular activities of the title compounds (**3a–3i**) were evaluated using the whole-cell screening methods; hence, it is somehow difficult to determine the molecular target that could be responsible for their observed activity. Nonetheless, different computational techniques were employed in an attempt to identify the putative macromolecular target(s) that could explain their probable mechanism of action. These efforts were driven by the fact that once the specific drug target is identified, prospective optimisation strategies towards designing more potent and selective drug candidates will be much easier and more rational.

To this end, we reviewed the literature to identify the various mycobacterial drug targets known to be essential for bacterial growth and survival. Next, we explored the PDB for available solved crystal structures for any of these essential targets. Our search revealed that there are many reported essential mycobacterial drug targets and many others were reported to be potential targets (Supplementary materials, Table S1). In this study, we selected 20 enzymes to serve as structural models in molecular docking studies (Table 2). The selection criteria of putative targets were based on the availability of the solved 3 D crystal structures of H37Rv MTB strain and the essentiality of the target for mycobacterial growth and survival. The selection of individual 3 D crystal structures was guided by considering the crystal resolution, being unmutated (wild type), overall quality of the crystal structures, and the absence of missing loops.

**Table 2.** The selected 20 essential mycobacterial drug targets that were used for molecular modelling studies.

Index	MTB Protein Target	Targeted pathway	PDB ID	References
1	Decaprenylphosphoryl‐β‐d‐ribofuranose oxidoreductase (DprE1)	Cell wall biosynthesis: arabinogalactan biosynthesis	4P8C	^92^^,^^93^^,^^94^
2	Enyol-ACP-reductase, (InhA)	Cell wall biosynthesis: mycolic acid biosynthesis	6R9W	^95^
3	Mycolic acid cyclopropane synthase (CmaA2)	Cell wall biosynthesis: mycolic acid biosynthesis	1KPI	^96^
4	β-ketoacyl acyl carrier protein synthase I (KasA)	Cell wall biosynthesis: mycolic acid biosynthesis	6P9L	^97^
5	Polyketide synthase (Pks13)	Cell wall biosynthesis: mycolic acid biosynthesis	5V3Y	^98^^,^^99^
6	Enoyl-CoA hydratase 6 (EchA6)	Cell wall biosynthesis: mycolic acid biosynthesis	5DUF	^100^
7	Transcriptional repressor of EthA monooxygenase (EthR)	Cell wall biosynthesis: mycolic acid biosynthesis (indirect)	5EYR	^101^
8	Alanine racemase (alr)	Cell wall biosynthesis: peptidoglycan biosynthesis	1XFC	^102^
9	MurE (Mur Ligase family)	Cell wall biosynthesis: peptidoglycan biosynthesis	2WTZ	^103^
10	Bifunctional enzyme (GlmU)	Cell wall biosynthesis	2QKX	^104^
11	2-methylcitrate synthase (PrpC) or (GltA3)	Fatty Acid Biosynthesis	3HWK	^105^
12	3-oxoacyl-[acyl-carrier-protein] synthase 3 (FabH)	Fatty acid biosynthesis	1HZP	^97^
13	β-ketoacyl-ACP reductase (MabA)	Fatty Acid Biosynthesis	1UZN	^106^
14	Aspartyl-tRNA Synthetase (AspS)	Protein synthesis	5W25	^107^
15	leucyl-tRNA synthase (LeuRS)	Protein synthesis	5AGS	^108^
16	Protein kinase B (PknB)	Signal transduction	5U94	^109^
17	Protein kinase A (PknA)	Signal transduction	6B2Q	^110^
18	Pantothenate kinase (PanK, type 1)	Cofactor biosynthesis: Coenzyme A biosynthesis	4BFZ	^111^
19	5′-pyridoxal phosphate (PLP)-dependent aminotransferase (BioA)	Cofactor biosynthesis: biotin biosynthesis	4XJO	^112^
20	Aspartate aminotransferase (aspAT)	Asp biosynthesis, and Asp-dependent nitrogen metabolism	6U7A	^113^

The crystal complexes of the selected targets were prepared, solvated, and minimised as detailed in the methods section. These preparations were performed in order to relax the complex and remove any possible distortions resulting from crystal packing^74^^,^^75^. Then, the binding site in each of the selected crystal structures was defined. Prior to docking the title compounds (**3a–3i**), the co-crystallized ligands were extracted and redocked into their respective enzyme’s active site in order to assess the definition of the binding site and the accuracy of the docking algorithm in reproducing the orientation of the native co-crystallized ligand. Moreover, the co-crystallized ligands (inhibitors) were intended to be used as virtual positive controls during the docking studies^76^. The used CDOCKER docking algorithm was successful in reproducing the native co-crystallized orientation of the redocked ligands with RMSD values ranging from 0.2 to 1.43 Å.

Once the docking protocol was validated, the compounds were prepared as stated previously, and docked into the active site of each selected enzymes. Out of the 20 selected enzymes, the compounds failed to dock into alanine racemase (alr), 2-methylcitrate synthase (PrpC), and aspartyl-tRNA Synthetase (AspS) enzymes.

The CDOCKER algorithm reports two types of scores which are forcefield based scores, the – CDOCKER energy (−CDE) and the – CDOCKER interaction energy (−CDIE). The former score accounts for ligand-target interactions along with internal ligand strain, the latter accounts only for ligand-target interactions. In structure-based drug design, of which molecular docking is most commonly used, the development of an efficient and accurate scoring function remains the main challenge^108^. Hence, it is recommended to rescore the docked ligands using different scoring functions to minimise any bias that could result from using a single scoring function. Broadly, scoring functions are classified into forcefield based, empirical, knowledge-based, and machine-learning-based scoring functions^108^. In this study, in addition to the two forcefield-based CDOCKER scores, the docked ligands were rescored using other empirical and knowledge-based scoring functions available in DS. Five empirical scoring functions, LigScore-1, LigScore-2, PLP-1, PLP-2, and Jain; in addition to two knowledge-based scoring functions, PMF and PMF04, were used to rescore the docked ligands’ poses (Supplementary materials S2). In DS, the output scores of all used scoring functions are reported as positive values, hence, the more positive the value the better the score and the better the binding affinity.

In order to identify the putative target(s) for the tested compounds, each of the different computational scores were compared with the experimentally determined MIC values to check the existence of any correlation between the two. This comparison was performed by calculating the Pearson Correlation Coefficient (*r*), which is a measure of the strength of a linear association between two variables, and it can take a range of values from +1 (positive correlation) to −1 (negative correlation), a value of zero indicates no correlation ^92^. Since the computational scores are reported as positive values, then, we were looking for negative correlation coefficients; that is, as the value of the computational score increases, the value of the MIC decreases (Table 3). Based on the correlation values, a macromolecular target that shows the highest negative correlation coefficient would be deemed a putative target for the tested compounds. The strength of association is determined by the value of *r*, in general, if |*r*| is 0.1 − 0.3 then there is a small association, 0.3–0.5 is medium, and 0.5–1.0 indicates large association between the two sets of variables.

**Table 3.** The overall correlation coefficients matrix between the MIC values of the compounds **3a–3i** and their computational scores for each of the selected target enzymes.

Index	Targets	LS1	LS2	−PLP1	−PLP2	Jain	−PMF	−PMF04	−CDE	−CDIE
1	CamA	0.13	**−**0.02	0.05	−0.25	−0.29	0.46	0.23	−0.04	−0.08
2	DprE1	−0.07	**−0.45**	0.17	−0.09	**−0.45**	−0.2	0.22	0.36	−0.21
3	FabH	0.16	−0.02	−0.1	0.01	**−0.33**	0.39	0.3	−0.02	−0.04
4	InhA	0.05	0.16	0.1	−0.03	−0.07	**−0.31**	−0.02	0.09	0.1
5	MabA	0.23	−0.08	0.18	0.16	−0.06	−0.14	0.22	−0.02	**−0.35**
6	LeuRS	0.67	0.43	0.33	0.51	0.22	0.01	0.21	0.38	0.31
7	GlmU	−0.09	0.19	0.28	0.29	0.16	−0.22	0.23	0.27	0.16
8	PanK	−0.25	**−0.33**	−0.24	−0.12	−0.1	0.2	0.16	0.14	0.05
9	PknB	0.46	0.34	0.54	0.48	0.01	−0.3	−0.12	0.01	0.08
10	PknA	0.25	0.47	**−0.32**	0.09	**−0.36**	−0.5	−0.37	−0.09	−0.05
11	KasA	−0.01	−0.08	0.24	0.2	0.18	0.24	0.23	−0.19	**−0.59**
12	**Pks13**	−0.38	0.13	0.6	0.43	**−0.39**	**−0.65**	−0.09	0.24	0.14
13	BioA	**−0.52**	0.24	0.37	0.03	0.17	−0.03	**−0.42**	0.47	0.36
14	AspAT	**0.35**	0.4	0.05	0.14	0.93	0.19	0.05	−0.09	−0.03
15	EchA6	0.34	0.23	−0.23	0.14	0.78	0.17	0.14	0.11	0.05
16	MurE	0.23	0.44	−0.21	0.09	0.12	−0.13	0.47	−0.09	0.12
17	EthR	0.46	0.1	0.18	0.22	0.11	0.15	0.05	0.16	0.08

The highlighted values in bold represent the highest correlation coefficient scores for each scoring function.

Table 3 represent the overall matrix of Pearson correlation coefficients based on all nine used scoring functions. The highest correlation coefficient between the MIC values and calculated scores was obtained with PMF scoring function when the compounds were docked into Polyketide synthase (Pks13) enzyme (*r* = −0.65). This high negative correlation means that active compounds, low MIC values, are correlated with high score. In other words, if the observed experimental activities are lined up with the computational scores (calculated binding affinities) of the designed compounds against one enzyme of the selected set, then, this enzyme could be the putative target that has been inhibited by the tested compounds. Accordingly, in our case, Pks13 is considered to be the putative target for our tested compounds.

Other important points that were considered when analysing the above results were; firstly, the identified putative target had also shown negative correlation values using other scoring functions (LS1 and Jain) relative to other enzymes. Secondly, the individual scores of the designed compounds were showing higher or comparable docking scores as compared to the redocked native co-crystallized ligand. Particularly, the relative docking (RD) scores^17^ of the most active compounds (compounds **3a**, and **3d**) were generally greater than or close to one; indicating that these compounds are expected to show good inhibitory potential against the putative target (Table 4). Once a putative target was identified, further analysis of the binding modes and binding interactions of the compounds was conducted by calculating their total free binding energies to Pks13-TE enzyme (Table 4).

**Table 4.** The top ranked docking scores of the compounds into Pks13 enzyme, and their top free binding energy scores, along with the relative docking (RD) score of the most active compounds (**3a** and **3d**).

Compounds		Docking Scores (Pks13)										MIC (µg/ml)	
Index	Name	LS1	LS2	−PLP1	−PLP2	Jain	−PMF	−PMF04	−CDE	−CDIE	TBE	HRv	MDR
1	**3a**	4.1	6.55	103.24	100.61	1.97	134.25	63.43	27.205	46.07	−12.47	8	16
2	**3b**	5.11	6.62	117.68	107.52	2.85	126.69	76.55	29.426	47.451	−14.73	32	32
3	**3c**	4.72	6.92	112.79	106.22	4.53	143.21	74.55	27.984	51.551	−8.15	11	NA
4	**3d**	5.17	6.94	121.61	109.44	4.42	138.38	100.74	29.526	49.428	−17.05	8	32
5	**3e**	4.87	7.09	125.76	116.1	4.26	122.99	79.43	36.391	53.449	−20.64	32	128
6	**3f**	3.74	6.87	125.62	110.37	2.35	118.28	75.77	31.371	49.573	−6.19	128	NA
7	**3g**	4.64	6.62	112.53	102.85	3.81	143.89	74.97	31.856	49	−14.66	32	NA
8	**3h**	3.97	6.9	119.25	107.77	2.64	133.66	71.53	30.655	49.581	−28.07	64	NA
9	**3i**	3.89	6.91	103.89	99.06	3.74	137.41	75.08	31.567	47.857	−18.80	11.3	NA
Co-crystallized ligand		5.25	7.01	133.07	129.46	6.87	106.85	80.85	27.53	56.49			
RD* score (3a)		0.78	0.93	0.78	0.78	0.29	1.26	0.79	0.99	0.82			
RD score (3d)		0.99	0.99	0.91	0.85	0.64	1.30	1.25	1.07	0.88			

*RD score = compound’s score/redocked co-crystallized ligand score.

#### 3.3.2. Analysis of the binding interactions of compound 3a with its putative target (Pks13)

In this study, the C-terminal thioesterase (TE) domain of the mycobacterial Polyketide synthase (Pks13) enzyme (Pks13-TE) has been identified as a putative target for the tested compounds. Biologically, the Pks13 enzyme catalyses the last condensation reaction of mycolic acid biosynthesis yielding an oxo‐mycolic acid intermediate which is then reduced to form a mature mycolic acid by a mycolyl reductase^97^^,^^98^^,^^114^. Mycolic acids, long α-alkyl-β-hydroxy fatty acids comprising 60–90 carbon atoms, are essential components of the mycobacterial cell wall and are also critical for mycobacterial persistence and pathogenesis^115^. The majority of mycolic acids are covalently bound to arabinogalactan-peptidoglycan forming the cell wall mycolyl-arabinogalactan-peptidoglycan complex. Moreover, they are associated with outer cell envelope lipids including trehalose monomycolate (TMM), trehalose dimycolate (TDM) and glucose monomycolate, also they can be found as free mycolic acids^116^^,^^117^. Given the prominent role of mycolic acids in mycobacterium cell viability and for virulence, enzymes involved in mycolic acids biosynthesis, such as Pks13, represent novel targets for drug development.

**Figure 2.**
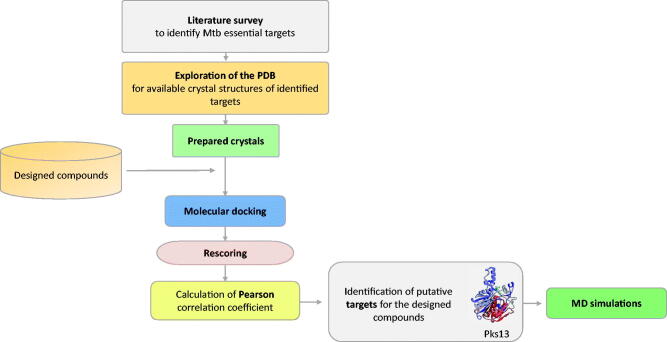
(A). The overall Pks13 domain structure. (B). Cartoon representation of the 3D crystal structure of the MTB Pks13 thioesterase domain (PDB code 5V3Y). The catalytic residues His1699 and Ser1533 located at the interface of the lid and core domains are depicted as ball and sticks with their carbons coloured green. The co-crystallized inhibitor is shown in ball and sticks with carbons coloured yellow. (C). Interpolated charge surface representation of the enzyme showing the large substrate binding groove, highlighted with dashed ellipsoid. (D). A close up view of the catalytic active site showing the catalytic triad.

Structurally, the Pks13 enzyme is comprised of five domains, a N-terminal acyl carrier protein (N-ACP), a β-ketoacyl-synthase (KS), an acyltransferase (AT), and a C-terminal acyl carrier protein (C-ACP), a C-terminal thioesterase (TE) domain. The topological structure of Pks13 has the order ACP-KS-AT-ACP-TE (Figure 2(A)). The structure of Pks13-TE domain comprises of a core domain and a lid domain. The core domain is the larger domain and consists of seven β-sheets (β1–β7), and four α helices (α1–α3 and α11), while the smaller lid domain consists of six α helices (α4–α9)^118^ (Figure 2(B)). The active-site pocket of Pks13-TE is located at the interface of the lid and core domains, and it harbours the catalytic triad of Ser1533, Asp1560, and His1699 (Figure 2(D)). The substrate binding pocket, the very long mycolic acid precursor carbon chains (C80–90), is a deep hydrophobic pocket extending from the active site spanning the full length of the lid domain (Figure 2(C))^118^. The co-crystallized inhibitor in the 5V3Y crystal complex binds in the fatty acyl chain-binding groove at the entrance of the active site, thereby, blocking substrate access to the catalytic binding site (Figure 2(C)).

**Figure 3.**
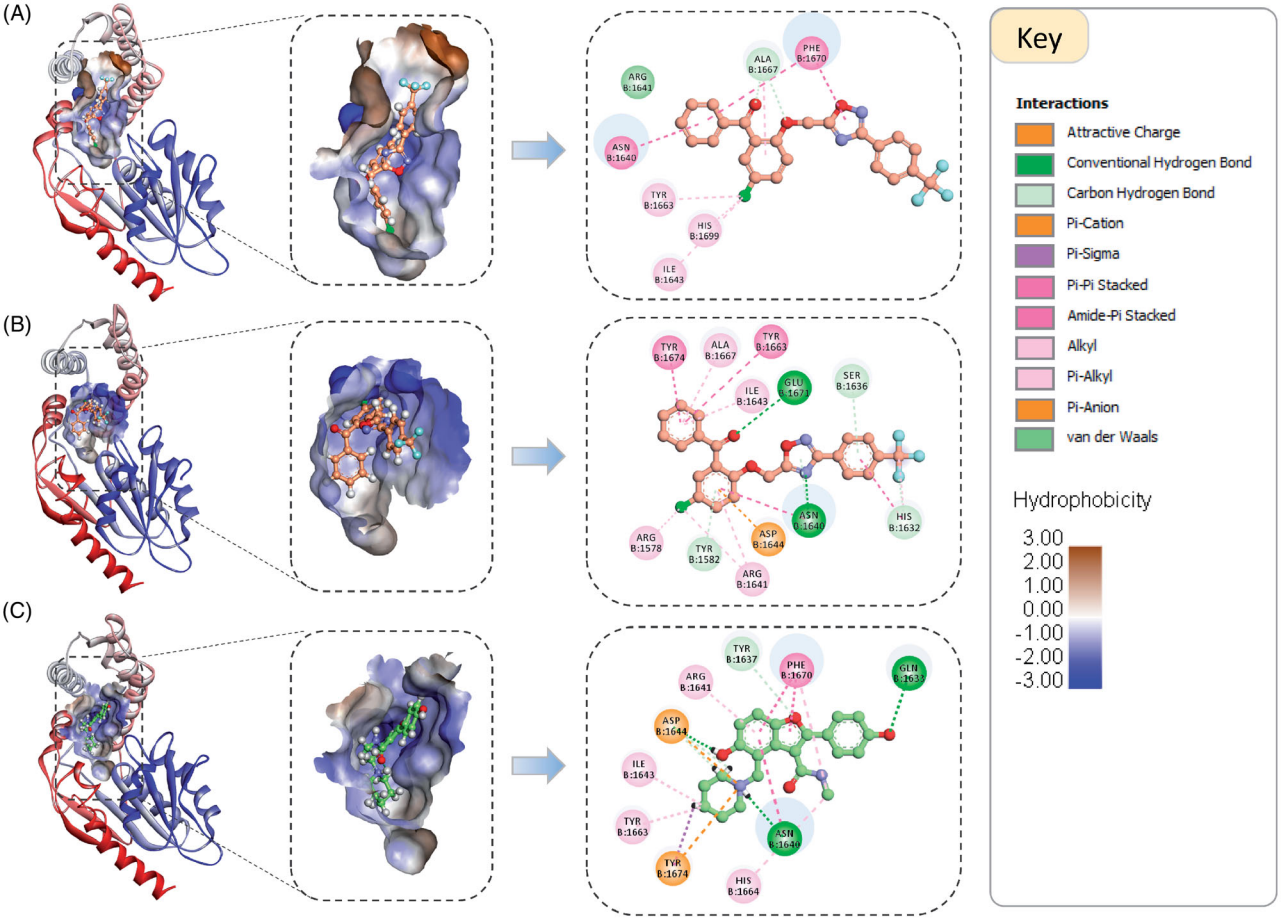
The binding orientation and binding interactions of compound **3a** within the binding groove of the Pks13-TE enzyme compared to the co-crystallized ligand (5V8, PDB code 5V3Y). (A). Top ranked docked pose of **3a** based on PMF score (PMF score = −134.3 kcal/mol, and TBE score = −6.9 kcal/mol). (B). Top ranked docked pose of **3a** based on free binding energy score (PMF score = −66.9 kcal/mol, and TBE score = −12.5 kcal/mol). (C). Binding orientation of the co-crystallized ligand. The left and middle panels shows the binding orientations of compound **3a** and 5V8 within the binding groove which is shown as hydrophobic surface. Right panel shows the 2 D interaction maps of the two compounds. The interacting amino acid residues are represented as discs coloured according to the type of interactions they are forming with the enzyme.

Visual inspection of the binding orientation and binding interactions of the docked compounds based on their PMF scores and total free binding energy scores revealed some discrepancies. For example, the top scoring pose of the most active compound (**3a**) based on PMF score is different from that based on TBE score, while the third ranked pose based on TBE score is matching the orientation of the PMF top scoring one. Although the PMF scores were showing the best correlation with experimental MIC values, the top scoring pose of compound **3a** based on TBE was better matching the pose of the co-crystallized ligand (Figure 3).

As shown in Figure 3(A), compound **3a** is fully occupying the substrate binding groove (fatty acyl chain-binding groove at the entrance of the active site), with its chlorophenyl moiety being oriented towards the catalytic site and forms a hydrophobic interaction with the catalytic His1699. In Figure 3(B), compound **3a** adopts a binding mode similar to that of the co-crystallized ligand and is forming very similar interactions with the same amino acid residues. Both of the binding modes of compound **3a** are reasonable and could compromise the accessibility of the binding site for the fatty acyl substrate. However, in reality one of these two possible binding modes should be favoured over the other. Therefore, further analysis is required to establish a better understanding of the binding modes of these compounds with their putative target.

The best computational method that can simulate the ligand binding to a target while accounting for full target and ligand flexibility and explicit solvent effect is MD simulations^93–95^. Therefore, MD was utilised to study the binding stability and interactions of a virtual complex of Pks13-TE and compound **3a**. Using MD is invaluable in gaining a better insight about the dynamical interaction behaviour of this virtual complex, ultimately, supporting or refuting the conclusion that Pks13-TE is the putative target for the designed compounds.

#### 3.3.3. MD Simulations of virtual complex of compound 3a with its putative target

**Figure 4.**
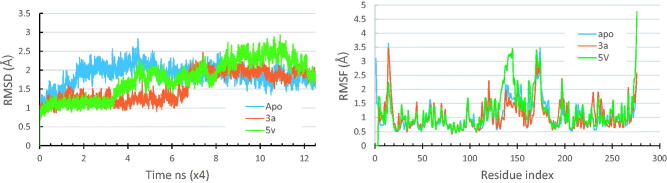
RMSD and RMSF plots for the simulated Pks13-TE-compound **3a** complex compared to those of the simulated apo form and that of the Pks13-5V8 complex.

MD simulations provide huge amount of dynamical, structural and energetic information about the simulated system^86^^,^^96^. The top ranked docked pose of the compound **3a** in Pks13-TE based on free binding energy scores was used as a structural model for running the simulations. Moreover, the apo forms of the enzyme, along with its solved crystal complexes (PDB code 5V3Y) were also simulated for comparison purposes. The apo forms of the Pks13 enzyme was modelled by deleting the co-crystallized ligand (5V8). The generated trajectories of all the simulated systems were analysed in terms of their thermodynamic properties, and their RMSD and RMSF. Figure 4 shows the RMSD and RMSF plots of all the simulated systems.

**Figure 5.**
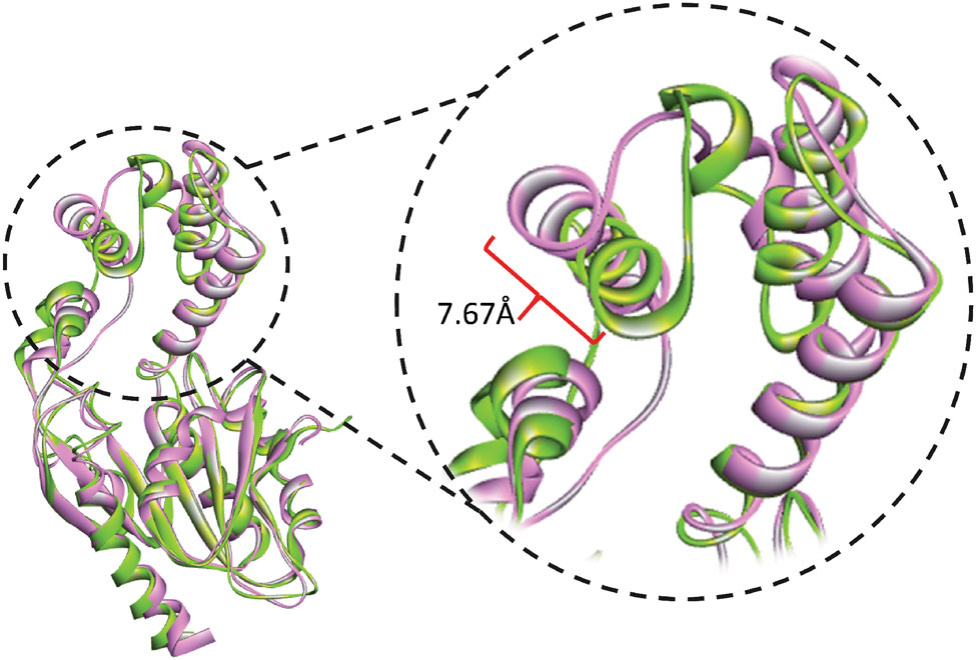
Cartoon representation of the conformational changes of simulated Pks13-TE-**3a** complex as it evolves with time. At 40 ns of simulation (green cartoon) helix 4 had moved inward by 7.67 Å relative to the conformation at 0 ns (pink cartoon).

The thermodynamic properties for all the simulated systems were quite stable throughout the simulation time. As shown in Figure 4, the RMSD values of backbone atoms of each of the Pks13 simulated complexes were smaller than that of the simulated apo form indicating that ligand binding had a stabilising effect on protein structure. The average RMSD values were 1.88 Å for the apo system, 1.01 Å for Pks13-**3a** system, and 1.77 Å for the Pks13-TE-5V8 system. Based on these values, compound **3a** shows an excellent stabilising effect, compared to 5V8, on the Pks13 enzyme which indicate that compound **3a** seems to have a good binding affinity to this enzyme. Moreover, the calculated RMSF of each simulated system, after being fitted to the first frame in order to get the fluctuations without rotations and translations, showed similar fluctuation patterns (Figure 4), with average values of 1.07 Å, 1.14 Å, and 1.16 Å for the apo system, Pks13-**3a** system, and Pks13-TE-5V8 system, respectively. The slightly higher RMAF values of the Pks13-TE-**3a** complex compared to that of apo system indicates that the latter seems to have experienced some sort of flexibility as a result of the induced fit to accommodate the change in binding orientation of the compound **3a**, and by the end of the simulation helix 4 had moved inward by 7.67 Å. (Figure 5).

**Figure 6.**
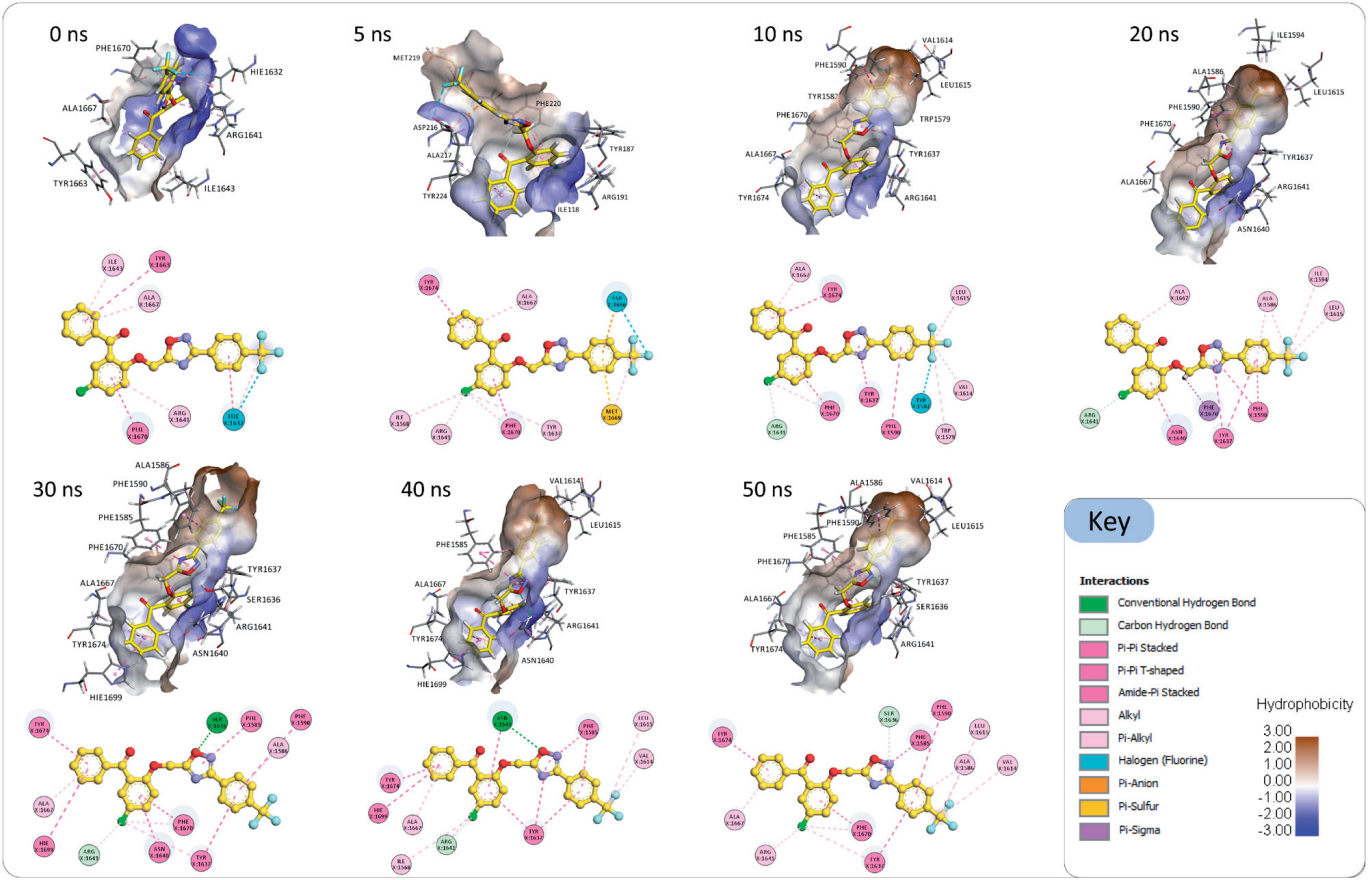
Snapshots of the simulated Pks13-TE-**3a** complex at 0 ns, 10 ns, 20 ns, 30 ns, 40 ns, and 50 ns, along with their corresponding 2 D interaction maps. The binding sites are shown as hydrophobic surfaces. Compound **3a** is shown in sticks with its carbon atoms coloured yellow. The 2 D interaction maps show the interactions of compound **3a** with Pks13-TE binding groove. Residues are coloured according to the type of their interactions with the ligand.

Visual inspection of the generated Pks13-TE-**3a** trajectory (Movie S1) clearly shows that compound **3a** had adjusted its binding orientation within the Pks13-TE binding groove during the first 7 ns to adopt an extended conformation that fully occupied the substrate binding groove and was maintained to the end of the simulation (Figure 6). Hence, MD simulations results add extra evidence that Pks13-TE enzyme is the most likely putative target for our designed compounds; yet, an *in vitro* enzyme assay will remain the main conclusive and decisive method to prove that the designed compounds have favourable binding affinity to mycobacterial Pks13-TE enzyme. Once the mechanism of action of our compounds have been confirmed as being mycobacterial Pks13-TE inhibitors, their detailed binding interactions can be utilised to guide the prospective optimisation of these lead compounds towards designing more potent and selective drug candidates.

As shown in Figure 6, after 7 ns of simulations compound **3a** has evolved into a binding orientation that is different from the initial one. This extended binding orientation was stabilised throughout the rest of the simulation time by establishing numerous interactions with amino acids in the substrate binding groove. The main stabilising interactions of the compound **3a** were Pi-Pi stacking and Pi-Pi T-shaped interactions with the aromatic amino acid residues Phe1585, Phe1590, Tyr1637, Phe1670, Tyr1674 and the catalytic His1699, and other hydrophobic interactions with Ala1586, Val1614, Leu1615, Arg1641 and Ala1667. Besides, there were intermittent hydrogen bonds with Ser1636 and Asn1640. Based on these interactions and its observed binding stability, compound **3a** is expected to have a favourable binding affinity towards the Pks13-TE enzyme that could be experimentally assessed via an *in vitro* enzyme assay.

#### 3.3.4. MD-Based docking

The MD simulations of the Pks13-TE-**3a** complex revealed that compound **3a** had evolved into a very stable binding state within the binding groove of the enzyme that was maintained throughout the simulation time. Therefore, the 3 D conformer of the enzyme in this stable state was used for a second run of molecular docking of the tested compounds in an attempt to explain their observed activities and establish a primary SAR analysis. The docking and rescoring runs were performed using the same protocol discussed above. However, the binding site in this run was defined based on the new orientation of the complexed compound **3a.** Table 5 shows the docking results and the Pearson correlation coefficient values for the different used scoring functions. It is evident that the individual docking scores, the relative docking scores, and most importantly the Pearson correlation coefficient had improved compared to the first docking results. A high correlation value of −0.81 between the MIC values and PMF scores confirms the existence of a strong linear association between the two variables, which in turn support our findings that Pks13-TE is most likely the putative target for our compounds (**3a–3i**).

**Table 5.** The top ranked docking scores of the tested compounds (**3a–3i**) into MD-based Pks13-TE conformer, along with the relative docking (RD) score of the most active compounds (**3a** and **3d**).

Compounds		Docking scores (Pks13)									MIC (µg/ml)	
Index	Name	LS1	LS2	−PLP1	−PLP2	Jain	−PMF	−PMF04	−CDE	−CDIE	HRv	MDR
1	**3a**	3.27	6.8	143.24	127.96	4.2	195.98	114.29	35.942	55.25	8	16
2	**3b**	3.39	6.49	131.41	115.83	3.59	187.11	104.12	31.375	52.05	32	32
3	**3c**	3.11	6.61	129.66	119.89	4.11	182.54	101.7	31.253	53.52	11	NA
4	**3d**	2.95	6.54	126.91	116.78	3.99	190.33	98.99	34.149	53.20	8	32
5	**3e**	3.13	6.68	128.13	111.94	4.19	165.49	90.75	31.519	55.47	32	128
6	**3f**	3.18	6.59	121.35	114.93	3.42	157.91	85.83	36.112	53.38	128	NA
7	**3g**	2.93	6.42	119.75	109.55	3.53	186.05	95.5	33.004	50.21	32	NA
8	**3h**	3.68	6.84	125.79	114.1	4.61	159.79	70.06	34.583	55.23	64	NA
9	**3i**	3.41	7.13	140.98	132.27	5.69	194.19	98.75	43.505	59.66	11.3	NA
Co-crystallized ligand		5.25	7.01	133.07	129.46	6.87	106.85	80.85	27.53	56.49		
RD* score (**3a**)		0.63	0.97	1.08	0.99	0.61	1.83	1.41	1.31	0.98		
RD score (**3d**)		0.56	0.93	0.95	0.90	0.58	1.78	1.22	1.24	0.94		
Pearson correlation		0.19	−0.16	**−0.58**	**−0.42**	**−0.39**	**−0.81**	**−0.64**	0.01	−0.18		

*RD score = compound’s score/redocked co-crystallized ligand score.

**Figure 7.**
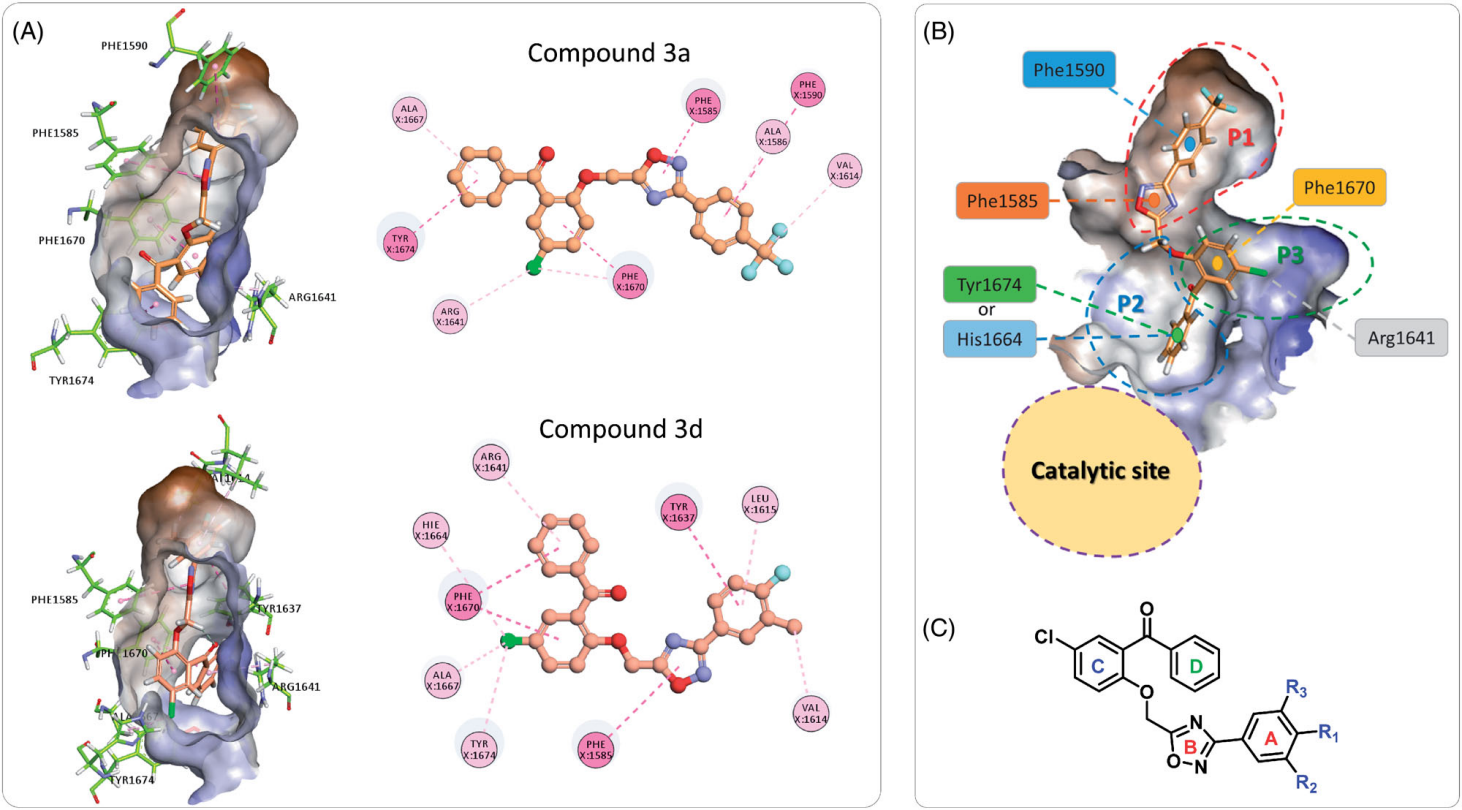
(A) The two binding modes of the designed compounds illustrated by the compounds **3a** and **3d**. For each compound, the 3 D binding mode is shown along with its 2 D interaction map, see Figure 5 for the key. (B) Detailed analysis of the Pks13-TE binding groove showing the catalytic site, and the substrate binding site with its three sub-pockets. (C) The general structure of the designed compounds.

The binding modes and binding interactions of the tested compounds as inferred from the second docking experiment in light with the topology of the Pks13-TE binding groove are quite consistent and can be distilled into two types (Figure 7(A)). The binding groove of Pks13-TE is divided into the catalytic site and the substrate binding site, the latter can be divided into three pockets (P1–P3) (Figure 7(B)). In both types of binding modes, the substituted phenyl ring (ring A) and the oxadiazole ring are occupying P1 hydrophobic pocket and are held in place by stacking with Phe1590 and Phe1585. The difference between the two binding modes is related to the orientation of rings C and D of the bound compound. In type one, ring D is fitting P2 pocket and is stacking with Tyr1674 and points towards the catalytic site; while ring C (chlorophenyl group) is occupying P3 pocket and is stacking with Phe1670 and is forming hydrophobic interaction with hydrophobic part of Arg1641 side chain. In type two binding, rings C and D switch binding pockets, such that ring C binds P2 pocket and ring D binds to P3 pocket. Either way, the substrate binding groove is fully occupied, hence, denying substrate access to the catalytic site.

### 3.4. ADME and toxicity predictions

The promising anti-tubercular activities of the tested compounds (**3a–3i**) are very encouraging to carry out further optimisation towards designing more drug-like candidates. Therefore, the ADME descriptors and toxicity parameters for these compounds were calculated to guide future optimisation and to focus on lead compounds that show favourable ADMET properties.

A range of ADME descriptors including, aqueous solubility (AS), blood brain barrier (BBB) penetration, CYP2D6 inhibition, hepatotoxicity, human intestinal absorption (HIA), plasma protein binding (PPB), AlogP, and polar surface area (PSA) were calculated using the ADMET Descriptors protocol in DS. Moreover, different toxicity parameters were also calculated using the Toxicity Prediction (TOPKAT) protocol including rodent carcinogenicity (for male and female rats and mice, CMR, CFR, and CLM, CFM respectively), Ames mutagenicity (AM), skin irritation (SI), ocular irritancy (OI), aerobic biodegradability and developmental toxicity potential (AB) (Table 6). The calculated ADMET properties presented in Table 6 indicate that compounds have promising ADME with no major toxicity profiles. Hence, the most active ones could be considered as leads worthy of further optimisation.

**Table 6.** Calculated ADMET descriptors and toxicity parameters of compounds (**3a–3i**).

Compounds		MIC (µg/mL)		ADMET descriptors^a^								Toxicity parameters^b^								
Index	Code	H37Rv	MDR-MTB	AS	BBB	CYP2D6 inhibition	Hepatotoxicity	HIA	PPB	AlogP	PSA	AM	SI	OI	AB	DTP	CMR	CFR	CLM	CFM
1	**3a**	8	16	1	4	TRUE	TRUE	1	TRUE	5.991	61.307	1	0.152	0.971	0	0	0.074	0	1	0.98
2	**3b**	32	32	1	1	TRUE	TRUE	0	TRUE	5.254	61.307	0	0	0.627	0	0	0.323	0	1	1
3	**3c**	11	No activity	1	4	TRUE	TRUE	1	TRUE	5.919	61.307	0	0	0.23	0	0	0.449	0	1	1
4	**3d**	8	32	1	1	TRUE	TRUE	1	TRUE	5.74	61.307	0	0	0.636	0	0	0.344	0	1	1
5	**3e**	32	128	2	1	TRUE	TRUE	0	TRUE	5.238	70.237	0	0	0.019	0	0	0.996	0.423	1	1
6	**3f**	128	No activity	1	4	TRUE	TRUE	1	TRUE	5.946	61.307	0	0	0.271	0	0	0.478	0.001	1	1
7	**3g**	32	No activity	2	1	TRUE	TRUE	0	TRUE	5.049	61.307	0	0	0.359	0	0	0.222	0	1	0.806
8	**3h**	64	No activity	2	1	FALSE	TRUE	0	TRUE	5.381	70.237	0	0	0.605	0	0	0.231	0	1	0.133
9	**3i**	11.3	No activity	1	4	TRUE	TRUE	1	TRUE	6.243	61.307	0	0	1	0	0	0.97	0	1	1

^a^Key to the above calculated ADMET descriptors							
Aqueous Solubility (AS)			Blood brain barrier (BBB) penetration			Human Intestinal Absorption (HIA)	
Level	Value	Drug-likeness	Level	Value	Description	Level	Description
0	log(Sw) < −8.0	Extremely low	0	Very High	Brain-Blood ratio greater than 5:1	0	Good absorption
1	−8.0 < log(Sw) < −6.0	No, very low, but possible	1	High	Brain-Blood ratio between 1:1 and 5:1	1	Moderate absorption
2	−6.0 < log(Sw) < −4.1	Yes, low	2	Medium	Brain-Blood ratio between 0.3:1 and 1:1	2	Low absorption
3	−4.1 < log(Sw) < −2.0	Yes, good	3	Low	Brain-Blood ratio less than 0.3:1	3	Very low absorption
4	−2.0 < log(Sw) ≤0.0	Yes, optimal	4	Undefined	Outside 99% confidence ellipse		
5	0.0 < log(Sw)	No, too soluble					

**^b^**Key to the above calculated toxicity parameters		
Probability values	Probability level	Description
0.0–0.30	Low probability	Such a chemical is not likely to produce a positive response in an experimental assay
Greater than 0.30 but less than 0.70	Intermediate probability	
Greater than 0.70	High probability	Likely to produce a positive response in an experimental assay

## 4. Conclusion

In the present work, anti-tubercular activity has been carried out for a series of 3,5-disubstituted-1,2,4-oxadiazole derivatives (**3a–3i**) against H37Rv, MDR and XDR strains of MTB. Compound **3a** with para-trifluorophenyl group attached to the 3-position of the oxadiazole ring was found to be the most active against the susceptible H37Rv and MDR strain of MTB with a MIC values of 8 µg/ml and 16 µg/ml, respectively. None of the compounds (**3a–3i**) showed any activity against the XDR strain.

To understand the mechanism of action as well as the probable drug target of these compounds (**3a–3i**), molecular docking studies were carried out against 20 mycobacterial enzymes reported to be essential for bacterial growth and survival. Docking studies revealed Polyketide synthase (Pks13, PDB code 5V3Y) enzyme as the probable target for these compounds. Further, molecular dynamics (MD) study was also carried out for the top ranked docked pose of the most active compound **3a** in Pks13. Moreover, the apo form of the enzyme (excluding the co-crystallized ligand 5V8), along with its solved crystal complexes (Pks13-5V8) was also simulated for comparison with the binding interaction of compound **3a**. Compound **3a** showed an excellent stabilising effect (RMSD for Pks13-**3a** system = 1.01 Å), as compared to 5V8 (RMSD for Pks13-5V8 system = 1.77 Å), on the Pks13 enzyme, indicating a good binding affinity of the compound **3a** towards the enzyme. The main stabilising interactions between compound **3a** and the enzyme were found to be π-π interactions with the aromatic amino acid residues Phe1585, Phe1590, Tyr1637, Phe1670, Tyr1674 and the catalytic His1699, including intermittent hydrogen bonding with Ser1636 and Asn1640. The 3 D conformer of the enzyme in this stable state (Pks13-TE-**3a** complex) was further used for a second run of molecular docking of all the compounds (**3a–3i**) in an attempt to explain their observed activities and establish a primary SAR analysis. A high correlation value of −0.81 (Pearson correlation coefficient) between MIC values and PMF scores confirmed the existence of a strong linear association between the two variables, thus reemphasizing Pks13 as the most likely putative target for the compounds **3a–3i**. *In silico* ADMET properties were also calculated to evaluate the pharmacokinetic properties and drug-likeness of these compounds. All the active compounds showed satisfactory ADME properties with no major toxic effects like tumorigenicity and mutagenicity. Thus these compounds, in particular compound **3a** can be considered as lead molecule for developing novel anti-TB agents.
